# Supplementary material for: An Indeterminate for Malignancy FNA Report Does Not Increase the Surgical Risk of Incidental Thyroid Carcinoma
Source: Cancers (Basel). 2022 Nov 3;14(21):5427. doi: 10.3390/cancers14215427 (PMC9657155; doi:10.3390/cancers14215427)
Supplement: Supplementary file 1 [file cancers-14-05427-s001.zip › cancers-1950847-supplementary.pdf]

Table S1. ITC series features (PTC: Papillary Thyroid Carcinoma; NIFTP: Non-Invasive Follicular Thyroid neoplasia with Papillary-like nuclear features).

N°	Bethesda cytological class	Age	Sex	Thyroid lobe	Diameter (mm)	Total or hemi-thyroidectomy	On target histological diagnosis	Ipsilateral carcinoma	Contralateral carcinoma	Single or multiple foci	Carcinoma diameter (mm)
1	III	68	F	Left	10	Total	Thyroid nodular follicular disease	Bilateral mPTC		Multiple	4
2	III	48	F	Right	35	Total	Thyroid nodular follicular disease	/	mPTC	Single	4
3	III	73	F	Left	35	Total	NIFTP	/	mPTC follicularvariant	Single	3
4	III	61	F	Left	35	Total	Thyroid nodular follicular disease	mPTC	/	Single	3
5	III	82	M	Right	40	Total	Thyroid nodular follicular disease	/	mPTC	Single	8
6	III	52	F	Right	10	Total	Chronic lymphocytis thyroiditis	/	mPTC	Single	9
7	III	50	F	Right	20	Total	Thyroid nodular follicular disease	mPTC follicular variant	/	Single	4
8	III	51	F	Right	10	Total	Thyroid nodular follicular disease and chronic lymphocytis thyroiditis	Bifocal mPTC follicular variant	/	Multiple	1
9	III	74	M	Left	30	Total	Thyroid nodular follicular disease	mPTC follicular variant	/	Single	1
1	IV	78	F	Left	39	Total	Oncocytic adenoma	Bilateral mPTC follicular variant		Multiple	4
2	IV	53	F	Left	30	Total	Oncocytic adenoma	/	mPTC	Single	2

Table S2. Demographic and clinico-pathological characteristics of incidental thyroid carcinomas according to the literature (AJCC: American Joint Committee on Cancer; FNAB: Fine Needle As-piration Biopsy; PTC: Papillary Thyroid Carcinoma).

PARAMETERS		SEMINATI et al. 2022	VASILEIADIS et al. 2013	BAHL et al. 2014	PEZZOLLA et al. 2014	FARRELL et al. 2016	GONZALEZ-SANCHEZ-MIGALLON et al. 2016	KALISZEWSKI et al. 2017	MATURO et al. 2017	EVRANOS et al. 2018	DE CARLOS et al. 2022
AJCC AGE GROUPS (years)	< 55	40%	35.7%	23.6%	/	/	Not applicable	26.7%	Not applicable	Not applicable	Not applicable
	≥ 55	60%	64.3%	76.4%				73.3%			
SEX	Male	28%	16.3%	30.3%	/	/	17.9%	14.7%	15.7%	25.5%	21.6%
	Female	72%	83.7%	69.7%			82.1%	85.3%	84.3%	74.5%	78.4%
HASHIMOTO'S THYRODITIS	Present	3%	/	/	/	/	/	/	/	34.2%	1.8%
	Absent	97%								65.8%	98.2%
GRAVES' DISEASE	Present	0%	/	/	/	/	/	/	/	8.6%	10.2%
	Absent	100%								91.4%	89.8%
FNA RESULT	Bethesda III	41%	/	/	/	Not applicable	/	/	/	91.1%	/
	Bethesda IV	59%								8.9%	
HISTOLOGY	Papillary (PTC)	100%	Not applicable	89.9%	97.5%	100%	/	82.7%	100%	100%	Not applicable
	Follicular	0%		2.2%	2.5%	0%		10.7%	0%	0%	
TUMOR SIZE (mm)	≤ 10	100%	Not applicable		57%	90.7%	51.2%	/	Not applicable	Not applicable	Not applicable
	11-19	0%		92.2%	43%	9.3%	48.8%				
	≥ 20			7.8%							
DOMINANT SITE	Right lobe	45%	/	/	/	/	/	/	/	/	/
	Left lobe	55%									
CANCER FOCI	1	64%	70.5%	/	/	79.1%	/	38.7%	84.3%	98.8%	12%
	≥ 2	36%	29.5%			20.9%		61.3%	15.7%	1.2%	88%
CAPSULAR INVASION	Present	5%	2.3%	/	/	/	/	/	/	7.9%	/
	Absent	95%	97.9%							92.1%	
VASCULAR INVASION	Present	0%	/	/	/	/	/	/	/	Not applicable	/
	Absent	100%									
EXTRATHYROIDAL EXTENSION	Present	0%	0%	/	0%	0%	/	4%	/	4.2%	3.6%
	Absent	100%	100%		100%	100%		96%		95.8%	96.4%
RESECTION	Complete	100%	/	/	/	/	/	/	/	98.2%	/
	Non-complete	0%								1.8%	
LYMPH NODE METASTASIS	Present	0%	4.7%	5.6%	2.5%	4.6%	/	2.6%	Not applicable	1.8%	0%
	Absent	100%	95.3%	94.4%	97.5%	95.4%		97.4%		98.2%	100%
DISTANT METASTASIS	Present	0%	0%	0%	0%	/	/	0%	Not applicable	0%	0%
	Absent	100%	100%	100%	100%			100%		100%	100%

**AJCC TUMOR STAGE**	I	100%	Not applicable	/	62.5%	/	85.1%	70.7%	Not applicable	/	/
	II				7.5%		8.3%	18.7%			
	III	0%			30%		3.6%	6.7%			
	**IV**				**0%**		**3%%**	**4%**			
